# Type I-F CRISPR–Cas provides protection from DNA, but not RNA phages

**DOI:** 10.1038/s41421-019-0123-9

**Published:** 2019-10-22

**Authors:** Murat Buyukyoruk, Blake Wiedenheft

**Affiliations:** Department of Microbiology and Immunology, Montana State University, Bozeman, MT 59717 USA

**Keywords:** Non-coding RNAs, Small RNAs

Dear Editor,

The type I-F CRISPR–Cas system from *Pseudomonas aeruginosa* (PA14) provides sequence-specific elimination of invading DNA^1,2^. Recently, a paper published in *Cell Research* reported that this system targets mRNA through a non-canonical mechanism^3^. Here, we implement the proposed design rules for generating CRISPR-RNA (crRNA)-guides that target RNA and test whether the proposed RNA-targeting activity provides immunity against RNA phage infection. Our experiments reveal that the type I-F CRISPR system provides protection from DNA, but not RNA phages.

The proposed mechanism of RNA targeting is distinct from dsDNA targeting. Rather than detection of a dsDNA PAM via protein-mediated recognition of specific nucleobases^4,5^, Li et al.^3^ suggest that single-stranded RNA recognition proceeds via recognition of a “PAM-like” motif (5′-GGN-3′) on the opposite side of the complex and that this is followed by detection of a 12-nt “core” sequence (Fig. 1a). To assess the versatility of these RNA-targeting rules, we designed I-F CRISPRs to target the RNA genome of MS2 phage^6^.

**Fig. 1:**
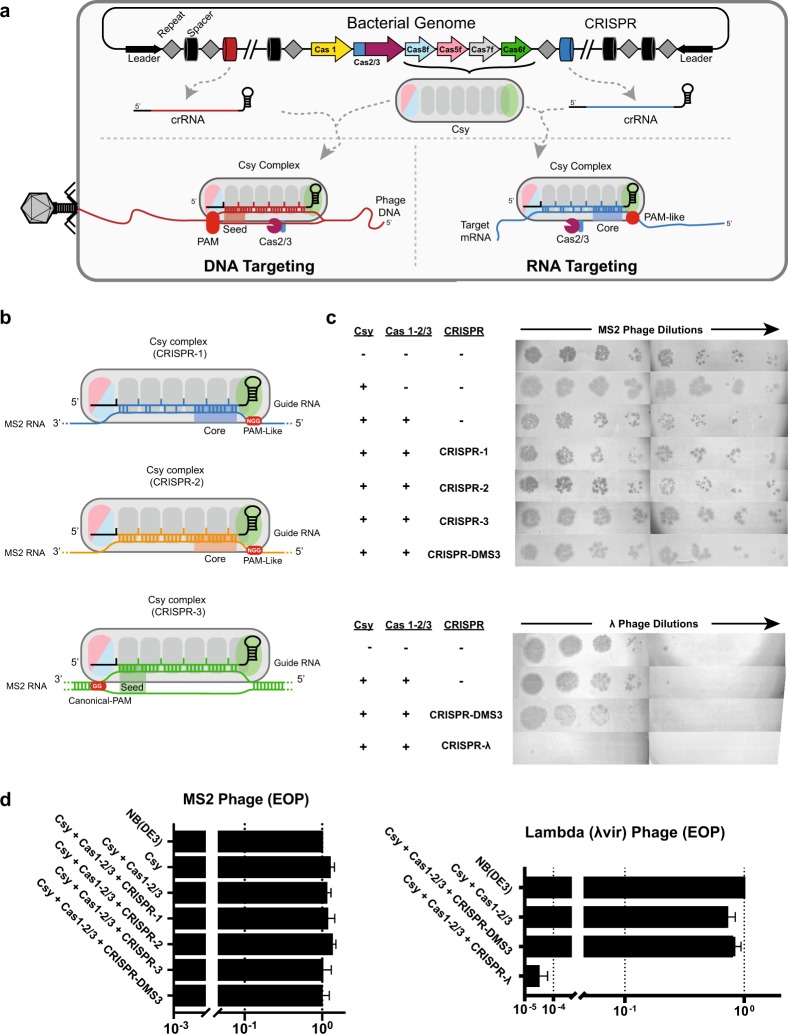
Testing the efficiency of type I-F CRISPR defense against RNA and DNA phages. **a** The type I-F CRISPR–Cas system of *P. aeruginosa* (PA14) contains six *cas* genes flanked by two CRISPR loci. DNA binding by the crRNA-guided surveillance complex (Csy complex) relies on recognition of a double-stranded PAM followed by a complementary protospacer. Base pairing occurs in discrete increments of five. Every sixth nucleobase is covered by a beta-hairpin on Cas7 and these bases do not contribute to target interactions^4,5^. Target binding drives a conformational change in the Csy complex that recruits the Cas2/3 nuclease for target cleavage. In contrast to dsDNA binding, the proposed RNA-binding model requires the recognition of a single-stranded “PAM-like” sequence followed by a 12-nt core sequence. **b** Schematics of CRISPRs 1, 2, and 3. CRISPR-1 (top) represents the model proposed by Li et al.^3^ CRISPR-2 (middle) targets the same sequences as CRISPR-1, but the crRNA-guide is completely complementarity. CRISPR-3 (bottom) was designed using well-established rules for dsDNA-binding mechanism. **c** Tenfold of dilutions of MS2 phage (top) or lambda (λ) phage (bottom) on a lawn of wild-type *E. coli* NB(DE3) cells or NB(DE3) expressing the type I-F CRISPR system. **d** The efficiency of plaquing (EOP) of MS2 phage (left) and lambda phage (right). EOP was calculated as the ratio of plaque-forming units (PFUs) on I-F CRISPR expressing cells divided by the number of PFUs on control cells

We designed three synthetic CRISPR arrays that each contains spacers designed to target six different regions in the MS2 RNA phage genome (Supplementary Fig. S1). Spacers in the first CRISPR (CRISPR-1) were designed to target sequences with a “PAM-like” NGG motif, a “core” sequence, and mismatches along the target sequence identical to those identified by Li et al.^3^ (Fig. 1b). Spacers in the second CRISPR (CRISPR-2), were designed to target the same RNA sequences, but the crRNA-guide is 100% complementary to the RNA target (i.e., no mismatches). Spacers in the third CRISPR (CRISPR-3) were designed according to established dsDNA targeting rules. Secondary structures in the MS2 genome are likely to be important for RNA targeting, so the six spacers included in each CRISPR were designed to target locations with varying degrees of predicted secondary structure. These structures are similar to secondary structures predicted for the *LasR* mRNA, which is purported to be targeted for degradation by the type I-F CRISPR system in PA14^3^.

To measure the efficiency of phage defense, we performed a series of plaque assays (Fig. 1c). None of the CRISPRs designed to target MS2 provided protection. To confirm that our experimental system is functional, we replaced the MS2 targeting CRISPR, with a CRISPR designed to target lambda (λ) phage (CRISPR-λ). Cells that express Csy, Cas2/3, and a synthetic CRISPR designed to target λ-phage, resulted in a 5-log reduction of plaques, as compared to non-targeting controls (i.e., CRISPR-DMS3) (Fig. 1c, d).

Importantly, the work presented here was not designed to replicate the previously published work by Li et al., but rather our intention was to determine if the non-canonical crRNA-guided recognition rules described by these authors could be generally applied for programable defense against RNA phages. Unlike the type III CRISPR systems described by Silas et al.^7^, the type I-F systems do not contain a reverse transcriptase, which would be necessary to incorporate new spacers from RNA-based parasites. Here, we by-pass new spacer acquisition, by creating synthetic CRISPRs designed to target the MS2 genome. Regardless of the design rule (canonical or non-conical), none of the MS2 targeting CRISPRs were capable of knocking down RNA phage infection. This biological result is supported by recent structural, biochemical, and biological assays which all show that Cas2/3 nuclease recruitment requires a conformational change in the Csy complex that is dependent on displacement of a non-complementary strand during crRNA-guided base pairing to the target DNA^4^. We acknowledge that CRISPR mechanisms are remarkably diverse and we remain open to alternative mechanisms for target recognition and Cas2/3 recruitment, however, Høyland-Kroghsbo et al.^8^ recently repeated the work by Li et al. but did not find evidence for RNA targeting in PA14. Collectively, these results suggest that type I-F CRISPR systems are capable of crRNA-guided detection and destruction of dsDNA, but not RNA.

## Supplementary information

Supplemental Material

## Supplementary information

**Supplementary Information** accompanies the paper at (10.1038/s41421-019-0123-9).
