# Supplementary material for: Type I-F CRISPR–Cas provides protection from DNA, but not RNA phages
Source: Cell Discov. 2019 Oct 22;5:54. doi: 10.1038/s41421-019-0123-9 (PMC6868142; doi:10.1038/s41421-019-0123-9)
Supplement: Supplementary file 1 — Supplemental Material [file 41421_2019_123_MOESM1_ESM.pdf]

Supplementary Materials and Methods

Strains, Plasmids and Growth Conditions

NovaBlue (DE3) *[endA1 hsdR17 (r_{K12}- m_{K12}+) supE44 thi-1 recA1 gyrA96 relA1 lac (DE3) F' $proA^+B^+$ lacIq ZΔM15::Tn10(Tet^R)]* cells were cultured at 37°C in liquid LB or on LB agar, supplemented with 0.1% glucose, 2mM $CaCl_2$ and 0.01 mg/ml thiamine. Plasmids used for Csy and Cas1-2/3 expression are available at AddGene (89232 and 89240, respectively)[1,2]. CRISPR-DMS3 is available at AddGene (89244)[1], while the other CRISPRs were designed, synthesized, and cloned into a PMK vector (**Supplementary Fig. S1**). CRISPR-λ contains four spacer sequences that target four Lambda phage genes (J, O, R and E). Growth media is supplemented with ampicillin to maintain the pCys1-4, spectinomycin to maintain pCas1-2/3, and kanamycin to maintain pCRISPR. Additionally, tetracycline is provided in the growth media for the selection of F' episome in NovaBlue (DE3). In NovaBlue (DE3) cells, the induction of expression vectors was achieved on LB Agar plates with 0.001mM isopropyl-D-1-thiogalactopyranoside (IPTG).

Synthetic CRISPR array construction

Three different CRISPR arrays was assembled that each contains six spacer sequences to target MS2 phage. Overlapping oligonucleotides (Eurofins Genomics) were annealed and extended at 72°C with Q5 polymerase to synthesize small fragments containing repeat-spacer-repeat. Small fragments were combined using overlap PCR extension and the final CRISPR was cloned using SacI and EcoRI sites (**Supplementary Fig. S1**). Each clone was sequenced (Genscript). Each CRISPR contains six spacers (32-bps) flanked by 28-bp repeats. Spacers were selected from a variety of locations on the MS2 genes based on the availability of the PAM or PAM-like region. CRISPRs were designed using the canonical model or non-canonical binding models proposed by Li *et al*[3].

Phage replication and Plaque Assays

E. coli BL21 (DE3) and K12 ER2738 used to propogagte Lambda_vir and MS2 phages, respectively. K12 ER2738 contains an F’ episome under tetracycline selection, which provides receptor for the MS2 phage attachment. 100mls of *E. coli* BL21 (DE3) and K12 ER2738 cells were grown to OD_{600} of 0.5 and then infected with 100µl of corresponding phage. Cultures containing phage were agitated overnight at

37°C. Cell lysates were collected by centrifugation (4000g for 30 minutes). A 0.2µm filter was used particulates from cell lysate. 100mls of the cell lysates were concentrated into 5ml and tittered by plaque assay. Tittering was performed using 100µl of *E. coli* cells grown to OD_{600} of 0.5. These cells were mixed with 100µl of phage or 10-fold dilutions of each phage, incubated for 30 mins and shaken every 10 mins. 5mls of molten 0.8% LB/agarose was added to the cell slurry and the soft agar was poured over the top of an LB-agar plate. Plates were incubated at 37°C overnight and plaques were counted the following day.

Spot Plaque assay

E. coli NovaBlue (DE3) cells were cultivated with appropriate antibiotics to maintain plasmids and challenge with Lambda_vir (dsDNA) (University of Laval, reference phage HER 37) or MS2 (+ssRNA) phages (ATCC 15597). NovaBlue (DE3) cells were grown to an OD_{600} of 0.9 and 400µl of the bacterial culture is mixed with molten 0.8% LB/agarose and poured over a solid agar. 5µl of each phage dilution were spotted on the top agar and the plates were incubated at 37°C overnight. Plates were evaluated the following day.

Quantification and statistical analysis

Efficiency of Plaquing (EOP)

Efficiency of plaquing (EOP) is reported as a ratio of PFUs formed on the experimental (i.e. CRISPR containing) strain divided by the number of PFUs on a wild-type. Plaque assays were performed in triplicate and the EOP is the ± standard deviation.

Supplementary Figure S1

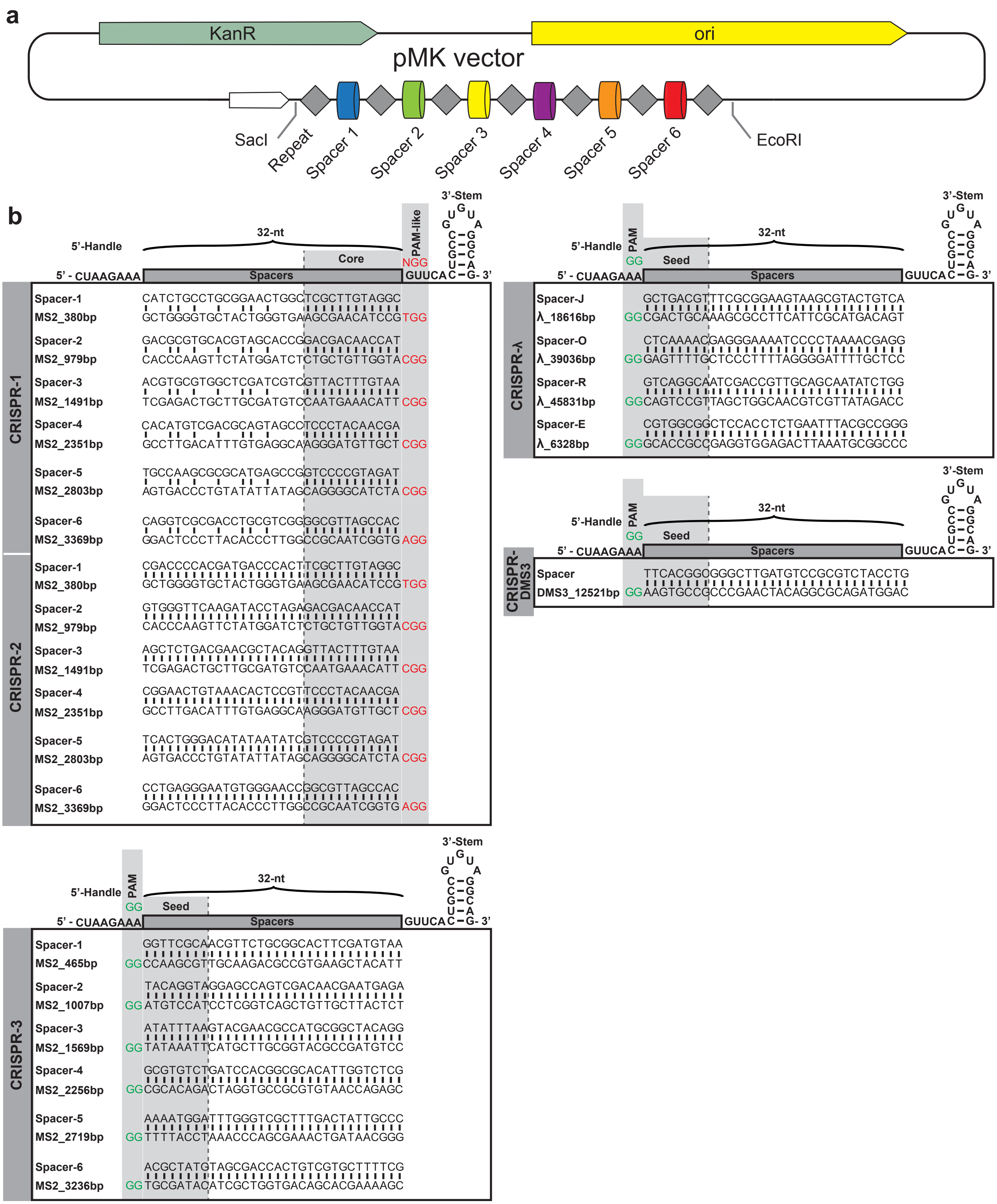

c

CRISPR-1 array
TAATACGACTCACTATAGGGCGAATTGAAGGAAGGCCGTCAAGGCCGCATGAGCTCGTTCACTGCCGTGTAGGCA
GCTAAGAAACATCTGCCTGCGGAACTGGCTCGCTTGTAGGCGTTCACTGCCGTGTAGGCAGCTAAGAAAGACGCGT
GCACGTAGCACCGGACGACAACCATGTTCACTGCCGTGTAGGCAGCTAAGAAAACGTGCGTGGCTCGATCGTCGTT
ACTTTGTAAGTTCACTGCCGTGTAGGCAGCTAAGAAACACATGTCGACGCAGTAGCCTCCCTACAACGAGTTCACTG
CCGTGTAGGCAGCTAAGAAATGCCAAGCGCGCATGAGCCGGTCCCCGTAGATGTTCACTGCCGTGTAGGCAGCTAA
GAAACAGGTCGCGACCTGCGTCGGGGCGTTAGCCACGTTCACTGCCGTGTAGGCAGCTAAGAAA

CRISPR-2 array
TAATACGACTCACTATAGGGCGAATTGAAGGAAGGCCGTCAAGGCCGCATGAGCTCGTTCACTGCCGTGTAGGCAG
CTAAGAAACGACCCCACGATGACCCACTTCGCTTGTAGGCGTTCACTGCCGTGTAGGCAGCTAAGAAAGTGGGTTC
AAGATACCTAGAGACGACAACCATGTTCACTGCCGTGTAGGCAGCTAAGAAAAGCTCTGACGAACGCTACAGGTTAC
TTTGTAAGTTCACTGCCGTGTAGGCAGCTAAGAAACGGAACTGTAAACACTCCGTTCCCTACAACGAGTTCACTGCC
GTGTAGGCAGCTAAGAAATCACTGGGACATATAATATCGTCCCCGTAGATGTTCACTGCCGTGTAGGCAGCTAAGAAA
CCTGAGGGAATGTGGGAACCGGCGTTAGCCACGTTCACTGCCGTGTAGGCAGCTAAGAAA

CRISPR-3 array
TAATACGACTCACTATAGGGCGAATTGAAGGAAGGCCGTCAAGGCCGCATGAGCTCGTTCACTGCCGTGTAGGCA
GCTAAGAAAGGTTCGCAACGTTCTGCGGCACTTCGATGTAAGTTCACTGCCGTGTAGGCAGCTAAGAAATACAGGT
AGGAGCCAGTCGACAACGAATGAGAGTTCACTGCCGTGTAGGCAGCTAAGAAAATATTTAAGTACGAACGCCATGC
GGCTACAGGGTTCACTGCCGTGTAGGCAGCTAAGAAAGCGTGTCTGATCCACGGCGCACATTGGTCTCGGTTCAC
TGCCGTGTAGGCAGCTAAGAAAAAAATGGATTTGGGTCGCTTTGACTATTGCCCGTTCACTGCCGTGTAGGCAGCT
AAGAAAACGCTATGTAGCGACCACTGTCGTGCTTTTCGGTTCACTGCCGTGTAGGCAGCTAAGAAA

CRISPR-λ array
GTTCACTGCCGTGTAGGCAGCTAAGAAAGCTGACGTTTCGCGGAAGTAAGCGTACTGTCAGTTCACTGCCGTGTAG
GCAGCTAAGAAACTCAAAACGAGGGAAAATCCCCTAAAACGAGGGTTCACTGCCGTGTAGGCAGCTAAGAAAGTCA
GGCAATCGACCGTTGCAGCAATATCTGGGTTCACTGCCGTGTAGGCAGCTAAGAAACGTGGCGGCTCCACCTCTG
AATTTACGCCGGGGTTCACTGCCGTGTAGGCAGCTAAGAAACAGGTAGACGCGGACATCAAGCCCGCCGTGAAAG
GTTCACTGCCGTGTAGGCAGCTAAGAAA

CRISPR-DMS3 array
TAATACGACTCACTATAGGGTTCACTGCCGTGTAGGCAGCTAAGAAATTCACGGCGGGCTTGATGTCCGCGTCTACC
TGGTTCACTGCCGTGTAGGCAGCTAAGAAATTCACGGCGGGCTTGATGTCCGCGTCTACCTGGTTCACTGCCGTGT
AGGCAGCTAAGAAATTCACGGCGGGCTTGATGTCCGCGTCTACCTGGTTCACTGCCGTGTAGGCAGCTAAGAAATT
CACGGCGGGCTTGATGTCCGCGTCTACCTGGTTCACTGCCGTGTAGGCAGCTAAGAAATTCACGGCGGGCTTGATG
TCCGCGTCTACCTGGTTCACTGCCGTGTAGGCAGCTAAGAAATTCACGGCGGGCTTGATGTCCGCGTCTACCTGGT
TCACTGCCGTGTAGGCAGCTAAGAAATTCACGGCGGGCTTGATGTCCGCGTCTACCTGGTTCACTGCCGTGTAGGC
AGCTAAGAAA

Supplementary Fig. S1. CRISPR design.

(a) Plasmid map of pMK vector containing six spacer sequences designed to target the RNA genome of MS2 phage. Each CRISPR array contains six (32-bps) spacers flanked by 28-bp repeats, cloned into the pMK backbone using SacI and EcoRI restriction sites. **(b)** Schematic of each crRNA mapped onto the target sequence. The flanking PAM-like (red) or PAM (green) sequences are indicated and

corresponding "core" or "seed" sequences are highlighted with a gray column. Each of the six spacers in CRISPR-1 and -2 target identical sequences that are flanked by a PAM-like motif, while CRISPR-3 spacers target proximal protospacers with a PAM. CRISPR-λ contains four spacer sequences that target four Lambda phage genes (J, O, R and E). CRISPR-DMS3 (negative control) contains seven identical spacers that target gene 24 in the DMS3 genome. **(c)** Sequences of the synthetic CRISPR loci. Spacers are separated by repeats (yellow). crRNA expression is under control of a T7 promoter (bold underline).

1. Chowdhury, S. *et al.* Structure Reveals Mechanisms of Viral Suppressors that Intercept a CRISPR RNA-Guided Surveillance Complex. *Cell* **169**, 47-57.e11 (2017).
2. Rollins, M. F. *et al.* Cas1 and the Csy complex are opposing regulators of Cas2/3 nuclease activity. *Proc. Natl. Acad. Sci. U.S.A.* **114**, E5113–E5121 (2017).
3. Li, R. *et al.* Type I CRISPR-Cas targets endogenous genes and regulates virulence to evade mammalian host immunity. *Cell Research* **26**, 1273–1287 (2016).
